# Robust Automatic Modulation Classification via a Lightweight Temporal Hybrid Neural Network

**DOI:** 10.3390/s24247908

**Published:** 2024-12-11

**Authors:** Zhao Wang, Weixiong Zhang, Zhitao Zhao, Ping Tang, Zheng Zhang

**Affiliations:** 1 Aerospace Information Research Institute, Chinese Academy of Sciences, Beijing 100094, China; 2 School of Electronic, Electrical and Communication Engineering, University of Chinese Academy of Sciences, Beijing 100049, China

**Keywords:** automatic modulation classification, temporal convolutional network, gate recurrent unit, lightweight model, hybrid model

## Abstract

In the rapidly developing field of wireless communications, the precise classification of modulated signals is essential for optimizing spectrum utilization and improving communication quality. However, existing networks face challenges in robustness against signals containing phase shift keying and computational efficiency. This paper introduces TCN-GRU, a lightweight model that combines the advantages of multiscale feature extraction of the temporal convolutional network (TCN) and global sequence modeling of gated recurrent unit (GRU). Compared to the state-of-the-art MCLDNN, TCN-GRU reduces parameters by 37.6%, achieving an accuracy of 0.6156 and 0.6466 on the RadioML2016.10a and RadioML2016.10b, respectively (versus MCLDNN’s 0.6101 and 0.6462). Furthermore, TCN-GRU demonstrates superior ability in distinguishing challenging modulations such as QAM16 and QAM64, and it improves classification accuracy by about 10.5% compared to MCLDNN. These results suggest that TCN-GRU is a robust and efficient solution for enhancing AMC in complex and noisy environments.

## 1. Introduction

Radio signals are a fundamental component of human communication. As communication technologies advance, channel environments have become increasingly complex, making accurate identification of various modulation methods within dense channels challenging due to noise and multipath fading. Automatic modulation classification (AMC) technology emerges as a critical solution in this context, serving as a vital intermediary between energy detection and the full demodulation process in decoding systems. AMC enhances spectrum efficiency, and it finds extensive application across diverse sectors. In commercial settings, AMC performs tasks like interference identification and frequency management, streamlining communication networks for better efficiency and reliability. In military contexts, AMC is indispensable for interception, jamming, and surveillance operations, offering strategic communication and signal intelligence advantages. The widespread applicability of AMC underscores its importance in advancing communication technology and supporting critical operational needs in various domains.

Originally, modulated signals were identified manually. Subsequently, AMC emerged, initially divided into likelihood-based (LB) and feature-based (FB) methods [1]. LB methods, grounded in mathematical rigor, offer optimal classification under Bayesian criteria but have high computational complexity and limited noise resistance [2]. FB methods use pattern recognition to map signal characteristics into feature spaces [3]. It utilizes prior knowledge for classifying signal modulation techniques, primarily through feature extraction including instantaneous aspects such as amplitude, frequency, and phase [4], higher-order statistics [5,6], transform domain characteristics involving Fourier and wavelet transform [7,8], as well as constellation [9]. Common classifiers of FB methods include support vector machines [10], decision trees [11], hidden Markov models [12], and k-nearest neighbor [13]. While effective, FB methods rely on manually extracted features, limiting their adaptability to complex, dynamic signals [14].

In complex electromagnetic environments, digital signals are vulnerable to noise interference. Elevated noise levels can obscure the information carried by the original signal, impacting the accuracy of modulation identification. The signal-to-noise ratio (SNR) is a critical metric to assess the extent of noise interference. It is defined by Equation (1):

(1) $$\mathrm{SNR}=10\log\frac{P_{s}}{P_{n}}$$

where $P_{s}$ denotes the signal power and $P_{n}$ signifies the power of the noise. A higher SNR indicates lesser noise content within the signal, while a lower SNR suggests increased noise levels. SNR falls below 0 dB indicating high noise interference. Accurate identification of modulation types is crucial for guiding demodulation in such cases.

Deep learning has demonstrated exceptional performance in fields such as image processing, speech recognition, and text analysis in recent years. This success has naturally extended to the field of AMC. Deep learning is data-driven. It adapts iteratively to input characteristics from large datasets, resulting in highly accurate, stable predictive models. Compared to LB and FB methods, it offers superior adaptability and excels at extracting complex features, particularly for the end-to-end classification of modulated signals at low SNR [15]. Currently, various deep network architectures including convolutional neural networks (CNNs) [16], recurrent neural networks (RNNs) [17], and a range of hybrid models are being used in AMC, each contributing uniquely to the advancement of AMC methodologies.

Networks based on CNN have shown impressive performance in image processing. O’Shea et al. employed a CNN model with two convolutional layers and two fully connected layers to classify 11 modulation formats of in-phase and quadrature (I/Q) sampled signals using the RadioML2016.10a dataset [18]. However, the relatively simple design of the network limited its ability to extract temporal features, resulting in an accuracy of only 57.0%. In contrast, Liu et al. [19] used the residual network (ResNet) [20] to analyze RadioML2016.10a and RadioML2016.10b. The accuracy on RadioML2016.10a was comparable to that of CNN, and the overall accuracy on RadioML2016.10b was able to reach 61.2% (compared to 59.4% for CNN), although it still fell short of the performance achieved by the hybrid network.

Fundamentally, AMC can be viewed as a time series modeling process. Therefore, many researchers have begun to study the effectiveness of RNN. Long short-term memory (LSTM) [21], known for its proficiency in modeling both forward and backward temporal dependencies, has been a focus in this area. Rajendran et al. [22] implemented a two-layer LSTM network to learn the amplitude and phase (A/P) characteristics. This method has an accuracy of nearly 87% for signals above 0 dB, but for signals below 0 dB, its classification ability is lower than that of the hybrid model [19,23] (about 2–4%). In addition, LSTM needs to be trained on a larger scale of data to achieve good results [15]. Gated recurrent unit (GRU) [24] has become a noteworthy alternative to LSTM. It provides similar modeling capabilities but with fewer parameters and faster training and prediction speed. Hong et al. [25] demonstrated the effectiveness of GRU in identifying I/Q signals in the RadioML2016.10a dataset. With RadioML2016.10a, GRU improves the classification accuracy of signals above 0 dB by about 4% compared to CNN and is similar to LSTM. It has an advantage in speed, with a training speed increase of about 14% compared to LSTM, which enhances its potential as a valuable tool in AMC.

The increasing complexity of wireless communication environments has posed significant challenges to accurately identifying signal modulation modes, particularly under low SNR conditions. Traditional methods such as LB and FB approaches, while effective in certain scenarios, suffer from drawbacks including high computational complexity, limited adaptability to noise interference, and inadequate feature extraction capabilities. Deep learning models, including CNNs and RNNs, have made strides in AMC, yet they often struggle to capture the full range of temporal dependencies or the intricate local–global feature relationships inherent in modulated signals.

Hybrid models, by integrating the strengths of CNNs and RNNs, offer superior results in modulation signal identification. This synergistic approach unlocks new potential in the realm of AMC. West et al. [26] integrating three one-dimensional convolutional layers with a single-layer LSTM. This model is faster than GRU and improved the classification accuracy by 1–2% over LSTM below 0 dB. Liu et al. [19] refined West’s model structure by incorporating a two-dimensional convolutional layer, denoted as convolutional long short-term deep neural network (CLDNN), enhancing its classification accuracy. Notably, the CLDNN model demonstrates superior performance to traditional CNN-type and RNN-type models such as ResNet, DenseNet [27], GRU, and LSTM, and its classification accuracy is more than 4–6% higher than previous models, being within −8–0 dB. This superiority underscores the effectiveness of combining CNN and RNN architectures in distinguishing features of different modulated signals. Advancing this approach, Xu et al. [23] proposed the multichannel CLDNN (MCLDNN). It utilizes three input channels to extract complementary features from the I/Q signal, with accuracies of approximately 61.0% and 64.6% for RadioML2016.10a and RadioML2016.10b, respectively (60.5% and 62.7% for CLDNN [19]).

Despite the significant advancements made by the aforementioned hybrid models in AMC compared to LB, FB, CNNs, and RNNs, they also have some limitations. Firstly, there is still room for improvement in the classification accuracy of hybrid networks, and some hybrid models [19,23,28] continue to face limitations when identifying signals with complex modulations, such as QAM and PSK. Both QAM and PSK use phase as the information carrier. As the signal’s SNR decreases, the phase’s change points are more easily masked by noise. The existing hybrid models generally use ordinary convolution to initially extract signal features without considering the multiscale features of QAM and PSK, which makes the classification accuracy relatively low. Additionally, these hybrid models often have complex network structures and numerous parameters, which require substantial computational resources and time during training and inference. To address these limitations, there is a need for a more robust network architecture that can effectively extract both the local fragmentary features and the overarching structural features of modulated signals, while also being computationally efficient.

In this paper, we introduce a novel hybrid neural network model, termed TCN-GRU. TCN-GRU was developed to overcome the challenges mentioned above by integrating the strengths of temporal convolutional networks (TCN) for multiscale feature synthesis and GRU for temporal sequence modeling, providing a more comprehensive solution to the limitations of previous methods. Then, TCN-GRU employs global average pooling (GAP) to fuse and map these features across the time scale. Finally, the classification result is obtained through a fully connected layer. The main contributions of this paper are as follows:

- The proposed TCN-GRU enables multiscale feature extraction and fusion, which allows our model to improve the classification accuracy of QAM and PSK, compared to other models;
- The proposed TCN-GRU is valid on RadioML2016.10a and RadioML2016.10b. Compared with some high-performing networks, our model demonstrates superior performance;
- The proposed TCN-GRU has fewer parameters than the existing high-precision model, and is structurally more efficient, making it highly practical.

## 2. Materials and Methods

In this section, we introduce the publicly accessible modulation signal datasets used to validate our model’s performance and their preprocessing methods. We also explain the architecture of the proposed TCN-GRU model and outline the evaluation metrics to assess its effectiveness and classification ability.

### 2.1. Modulation Signal Datasets

This study use the RadioML2016.10a and RadioML2016.10b datasets from DeepSig [29] to train and evaluate various deep network models. These datasets include numerous signal samples across multiple modulation methods and simulate real-world electromagnetic conditions by incorporating additive white Gaussian noise, multipath fading, center frequency offset, and sample rate offset. The details of these datasets are provided in Table 1.

RadioML2016.10a and RadioML2016.10b consist of I/Q signals with 128 sample points. However, they can also be represented in A/P format. Previous research [22] has shown that data in A/P format can provide more intuitive physical features, thereby improving the model classification accuracy. The conversion from I/Q to A/P representation is described by Equations (2) and (3) as follows:

(2) $$X_{A}=\sqrt{X_{I}^{2}+X_{Q}^{2}}$$

(3) $$X_{P}=\frac{2}{\pi }\arctan\frac{X_{I}}{X_{Q}}$$

where $X_{I},\;X_{Q}$ represent the I and Q channel signal sequences, respectively, and $X_{A},\;X_{P}$ denote the amplitude sequence and the normalized phase sequence, respectively.

In our experiments, RadioML2016.10a and RadioML2016.10b were randomly divided in an 8:1:1 ratio using a seed of 114,514. Of these, 80% of the signals (176,000 for RadioML2016.10a and 960,000 for RadioML2016.10b) is used for training, 10% (22,000 and 120,000) is used for validation—where validation loss determined model saving—and the remaining 10% is used for testing. The signal quantities for each modulation type and SNR level are uniformly distributed across all sections.

Using these two datasets to validate the model helps evaluate its convergence and robustness. RadioML2016.10a provides a baseline test covering a wide range of modulation types, and a smaller number of samples can test the convergence of the model. RadioML2016.10b has a larger number of data, which can evaluate the robustness of the model. Using two datasets to evaluate the model can more comprehensively reflect the classification ability of the model.

### 2.2. Deep Learining Networks

#### 2.2.1. Temporal Convolutional Network

**Figure 1:**
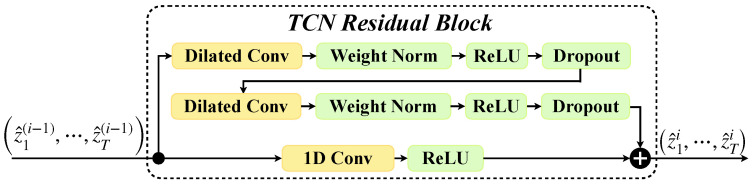
TCNRB is the foundational module of TCN. It can accommodate arbitrary lengths of inputs while ensuring equal length for both input and output. TCNRB is also characterized by its flexible receptive field size [30].

TCN is a stable and efficient model for time series prediction [30]. Similar to RNNs, TCN is stackable, handles arbitrary input lengths, and maintains equivalent lengths for input and output. To manage varying input lengths, TCN uses dilated convolutions [31,32], designed to capture temporal relationships by ensuring the output at time *t* depends solely on inputs up to the past-time point t−1. For equal input–output lengths, TCNs use a 1D fully convolutional network architecture [33] with zero padding. TCN integrates these functionalities with residual blocks (TCNRB), facilitating deep stacking of layers while preserving the receptive field size of dilated convolutions. The architecture of TCNRB is depicted in Figure 1.

TCN has a flexible receptive field size, enabling it to handle time series of various lengths through dilated convolutions and residual structures. Dilated convolutions expand the receptive field exponentially via the dilation factor, significantly increasing the receptive field without adding network depth. This enables TCN to capture features at multiple scales, reducing overall depth while effectively processing time series of varying lengths. For one-dimensional time series $x\in R^{n}$ and filter f:{0,1,⋯,k−1}→R, the operation process of dilated convolution is shown in Equation (4):

(4) $$F\left(s\right)=\sum_{i=0}^{k-1}f\left(i\right)\cdot x_{s-d\cdot i}$$

where *d* represents the dilation factor, *k* denotes the kernel size, and s−d·i stands for the timestamp of the past. The size of the receptive field *R* is calculated with Equation (5):

(5) $$R=1+2\cdot \left(k-1\right)\cdot N\cdot \sum_{i=0}^{N}d^{i}$$

where *k* represents the size of the dilated convolution kernel in TCNRB, *N* represents the number of TCNRB layers, and $d^{i}$ represents the dilation coefficient of the *i*-th layer residual block, with *d* typically sey to 2.

#### 2.2.2. Gate Recurrent Unit

**Figure 2:**
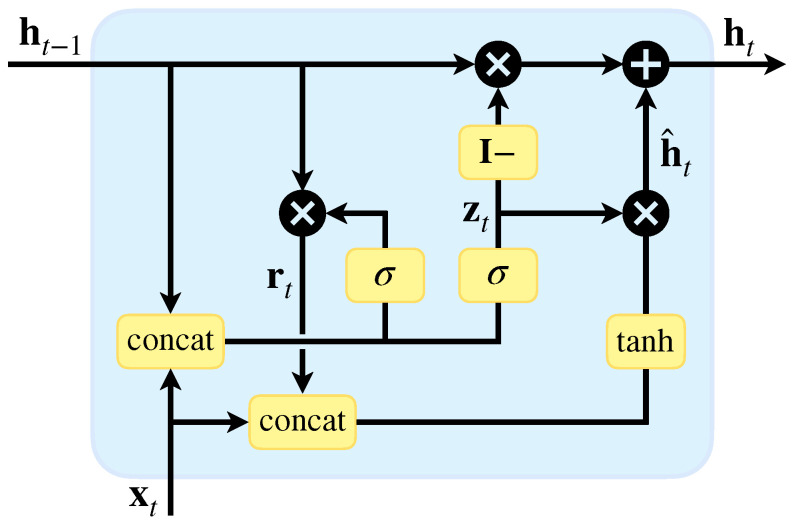
The structure of the GRU cell, which includes only two gates, taking into account both long-term and short-term memory.

GRU [24] simplifies the LSTM architecture. It combines the forget gate and input gate into a single update gate, streamlining the network unit. The GRU cell is illustrated in Figure 2, with forward propagation governed by Equations (6)–(9). This design makes GRU an efficient alternative to LSTM for sequence modeling tasks.

At each time step *t*, the input signal $x_{t}$ and the previously hidden state output $h_{t-1}$ are fed into the GRU’s reset gate $r_{t}$ and update gate $z_{t}$. These gates utilize the Sigmoid activation function, which outputs values between [0,1]. A value of 1 indicates full retention of new information, while 0 implies complete disregard. The reset gate determines how much past information is forgotten, while the update gate decides how much is retained. Next, the Hadamard product (denoted as ⊙) of $r_{t}$ and $h_{t-1}$ captures relevant past information to create the candidate hidden state ${\hat{h}}_{t}$, processed by the tanh function to mitigate gradient issues. Finally, the update gate controls the mix of memory from $h_{t-1}$ and the new information from $h_{t}$, as well as the amount of new information from ${\hat{h}}_{t}$ in the current output, allowing the GRU to balance past and new information effectively. These are calculated as follows:

(6) $$r_{t}=\sigma \left(W_{r}\cdot \left[h_{t-1},x_{t}\right]+b_{r}\right)$$

(7) $$z_{t}=\sigma \left(W_{z}\cdot \left[h_{t-1},x_{t}\right]+b_{z}\right)$$

(8) $${\hat{h}}_{t}=\tanh\left(W_{h}\cdot \left[r_{t}⊙h_{t-1},x_{t}\right]+b_{z}\right)$$

(9) $$h_{t}=\left(1-z_{t}\right)⊙h_{t-1}+z_{t}⊙{\hat{h}}_{t}$$

#### 2.2.3. TCN-GRU

Modulation signals exhibit diverse characteristics, and some related modulation modes may have highly similar time-domain features. A single network often faces limited classification accuracy for complex or similar modulated signals. Hybrid networks combine different network types in series and leverage the strengths of multiple networks. This synergy allows for more effective feature extraction and capturing the diverse characteristics of modulated signals in various forms. As a result, hybrid networks can overcome some of the feature recognition limitations inherent in single-network models. Our proposed TCN-GRU model combines two temporal feature extraction networks, i.e., TCN and GRU, resulting in a simpler structure with fewer parameters than many hybrid networks. In addition, TCN-GRU offers stronger feature extraction capabilities than CNN-type, RNN-type, and some hybrid models, making it effective for AMC.

Our TCN-GRU model consists of four TCNRBs and two GRU layers. Each TCNRB uses a convolution kernel size of 1×7 and contains a hidden size of 64. The GRU layers also have a hidden size of 64.

**Figure 3:**
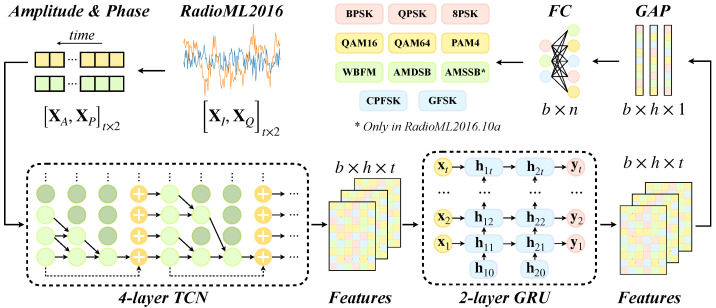
The whole process of AMC in this study. The TCN network, comprising a four-layer TCNRB, a two-layer GRU, and a GAP, is engineered to discern the multiscale and temporal features of AP complex signals for effective AMC.

The process of the TCN-GRU in classifying modulated signals is shown in Figure 3. The 2×128 I/Q signal from RadioML2016 is first transformed into a 2×128 complex signal in A/P form. These A/P signals then are fed into the TCN-GRU network. The TCNRBs progressively increase the receptive field, capturing local features at shallow layers and broader structural features at deeper layers. These feature maps are then processed by the GRU layers, which extract deeper, overarching temporal features of the signal. We employ GAP to fuse multiscale and temporal sequence features. It can also reduce feature map dimensions and model parameters, summarize time-dimension information, and speed up training. Finally, a fully connected layer is used to classify the extracted features, producing predicted probabilities for various modulated signals. This structured approach enables TCN-GRU to efficiently and accurately identify modulation types in complex signals. For optimization, we employ cross-entropy loss to quantify the deviation between predictions and the ground truth, and the Adam optimization method [34] is used for updating network parameters.

### 2.3. Performance Metrics

To evaluate the models’ capability in identifying various modulated signals, four evaluation metrics are employed: Accuracy, Precision, Recall, and F1-Score. These metrics provide a comprehensive understanding of the models’ performance, reflecting different aspects of classification accuracy and reliability. The relationships between prediction and ground truth are defined as follows:

- True Positive (TP): Positive samples correctly predicted as positive;
- True Negative (TN): Negative samples correctly predicted as negative;
- False Positive (FP): Negative samples incorrectly predicted as positive;
- False Negative (FN): Positive samples incorrectly predicted as negative.

For an N-class classification problem, the Accuracy metric represents the ratio of correctly predicted samples to the total number of samples in the test set, measuring the probability of accurate predictions. For the *i*-th class, the calculation method of Accuracy is detailed in Equation (10):

(10) $$\mathrm{Accuracy}=\frac{\sum_{i=1}^{N}\left({\mathrm{TP}}_{i}+{\mathrm{TN}}_{i}\right)}{\sum_{i=1}^{N}\left({\mathrm{TP}}_{i}+{\mathrm{TN}}_{i}+{\mathrm{FP}}_{i}+{\mathrm{FN}}_{i}\right)}$$

Precision indicates the proportion of actual positive samples among all samples predicted as positive, making it vital when the consequence of FP is significant. Conversely, Recall reflects the proportion of positive samples correctly predicted. It becomes crucial when the cost of FN is high. The calculation methods for Precision and Recall are detailed in Equations (11) and (12):

(11) $$\mathrm{Precision}=\frac{1}{N}\sum_{i=1}^{N}\frac{{\mathrm{TP}}_{i}}{{\mathrm{TP}}_{i}+{\mathrm{FP}}_{i}}$$

(12) $$\mathrm{Recall}=\frac{1}{N}\sum_{i=1}^{N}\frac{{\mathrm{TP}}_{i}}{{\mathrm{TP}}_{i}+{\mathrm{FN}}_{i}}$$

Precision and Recall measure the model’s ability to differentiate between negative and positive samples. Typically, a model with high Precision tends to have a lower Recall and vice versa, reflecting an inherent trade-off between these metrics. F-Score is the weighted harmonic average of Precision and Recall, providing a balanced measure of the model’s robustness by considering its performance on both positive and negative samples. Equation (13) presents the formula for calculating the F-Score:

(13) $$F\text{-}\mathrm{Score}=\left(1+\beta^{2}\right)\cdot \frac{\mathrm{Precision}\cdot \mathrm{Recall}}{\beta^{2}\left(\mathrm{Precision}+\mathrm{Recall}\right)}$$

When β=1, the F-Score becomes the F1-Score, indicating that Precision and Recall are given equal weight. In the context of an N-class classification problem, the calculation of the F1-Score is presented in Equation (14):

(14) $$F1\text{-}\mathrm{Score}=\frac{1}{N}\cdot \left(\sum_{i=1}^{N}2\cdot \frac{{\mathrm{Precision}}_{i}\cdot {\mathrm{Recall}}_{i}}{{\mathrm{Precision}}_{i}+{\mathrm{Recall}}_{i}}\right)$$

## 3. Experiments and Results

In the following experiments, we first assess the proposed models’ performance under various parameter configurations. After identifying the optimal parameters, we conduct comparative and ablation studies using RadioML2016.10a and RadioML2016.10b. The models are evaluated across three dimensions: overall performance indices, overall accuracy at each SNR level, and accuracy for each type of modulated signal. All experiments are conducted on a graphics workstation with an Intel Xeon W-2245 CPU and an NVIDIA Quadro RTX5000 GPU, providing robust computational support for training and analyses.

### 3.1. Determination of Network Structure

TCN and GRU are stackable neural networks. Their performance is influenced by factors such as the number of layers, hidden size, and the convolutional kernel size. To find the optimal configuration with the highest accuracy, we designed a series of experiments using RadioML2016.10a to identify the most effective architecture.

1. Layer Optimization: We evaluated the valid F1-Score of networks with varying numbers of TCN and GRU layers, keeping hidden size and convolution kernel size constant. This determined the optimal layer count for both TCN and GRU.
2. Hidden size Optimization: With the TCN and GRU layers fixed, we assessed the valid accuracy for different hidden sizes in TCN and GRU, maintaining a constant kernel size to identify the ideal count for each network.
3. Convolution Kernel Size Optimization: Based on the optimal number of layers and hidden size, we evaluated the valid accuracy for networks with varying TCN convolution kernel sizes finalizing the network structure.

**Figure 4:**
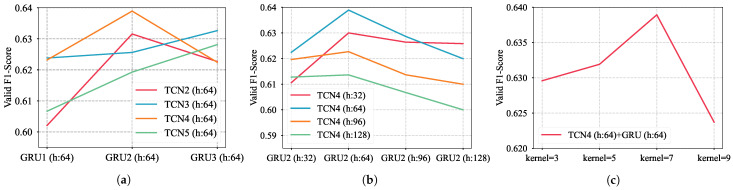
Three comparative experiments are designed to find a set of parameters that give the model the highest recognition accuracy. The results of these three experiments are: (**a**) comparison between layers, (**b**) comparison between hidden size, and (**c**) comparison between kernel size.

The comparative results are shown in Figure 4. The results indicate that a network with a four-layer TCN (kernel size of 7, 64 hidden size) combined with a two-layer GRU (64 hidden size) enhances the model’s capability to extract signal A/P features, resulting in higher accuracy.

### 3.2. Comparison of Performance Between Models

We conduct a performance comparison to evaluate TCN-GRU against some established methods in AMC, including CNN-type models like CNN2 [18] and ResNet [19], RNN-type models such as LSTM2 [22] and GRU2 [25], and hybrid models like CLDNN [19], MCLDNN [23], and CGDNet [35]. Notably, MCLDNN is the current state-of-the-art (SOTA) method in this field.

We also conduct ablation experiments to validate the efficacy of TCN-GRU. The control groups include GRU2-64: wop (two-layer GRU with 64 hidden size), GRU2-64 (two-layer GRU with 64 hidden size + GAP), TCN4: wop (four-layer TCN with 64 hidden size), TCN4 (four-layer TCN with 64 hidden size + GAP), and TCN-GRU: wop (TCN-GRU removed GAP). These comparisons demonstrate that the integration of TCN, GRU, and GAP achieves superior performance.

The structures of all networks are detailed in Table 2, where *c* denotes the number of channels, *k* the kernel size, *N* the prediction categories (11 for RadioML2016.10a and 10 for RadioML2016.10b), and *h* the hidden size. Conv refers to convolutional layers, FC to fully connected layers, Concat to concatenation, and MaxPool to max pooling.

The comparison and analysis of different network models encompass three main dimensions. They are:

1. Performance metrics: Each model’s performance is compared using the metrics outlined in Section 2.3, aiming to reflect the classification capabilities of each model.
2. Overall accuracy (OA) in different SNRs: We assess and compare the OA of all models across a spectrum of SNRs, measuring the model robustness in various noise powers.
3. Confusion matrices: We use confusion matrices to evaluate the model’s ability to recognize different modulated signals.

To mitigate the impact of high-power noise, we specifically compare each model’s OA within an SNR range of −8 dB to 18 dB. This targeted comparison helps us understand performance under common noise conditions.

#### 3.2.1. Comparison Between Model Performance Metrics

We use seven metrics to compare model performance: the number of parameters, validation cross-entropy loss, Accuracy, F1-Score, Recall, Precision, and prediction time on the test set. Prediction time is the average time to predict 22k samples (RadioML2016.10a) and 120k samples (RadioML2016.10b) taken by the model over five trials. The quantitative comparison results on both datasets are shown in Table 3 and Table 4.

In comparison experiments, we observed that CNN-type models (CNN2 and ResNet) have difficulty completing the AMC task, showing relatively low classification accuracy on both datasets. While fast in prediction, their high parameter count limits storage and loading speed. RNN-type models (GRU2 and LSTM2) achieved high prediction speeds. These models underperformed with limited samples but improved with larger datasets. Hybrid models like CGDNet, CLDNN, MCLDNN, and the proposed TCN-GRU exhibited distinct advantages. CGDNet excelled in speed. CLDNN, despite having numerous parameters, slightly outperformed CNN-type and RNN-type models on both datasets. MCLDNN displayed high accuracy on both datasets, outperforming other networks. TCN-GRU surpasses all others in multiple evaluation metrics, with a 37.6% reduced parameters compared to MCLDNN, making it less prone to overfitting in small-sample scenarios. Besides, TCN-GRU demonstrates a more significant accuracy improvement on RadioML2016.10a, which also highlights its strong generalization capability across small sample sizes. The ablation experiment results show that the TCN-GRU outperforms all control models on both datasets, underscoring the effectiveness of our proposed architecture and the synergistic advantages of combining TCN and GRU components.

TCN-GRU outperforms the current SOTA method in storage and loading efficiency. It is noticable that TCN-GRU’s computing speed is slightly lower, positioning it as a more storage-efficient option. This reflects a trade-off between prediction speed and accuracy, with our hybrid model prioritizing accuracy at a slight cost to inference speed. Despite this, the model’s reduced parameters lower overfitting risks and enhance generalization, making it highly suitable for practical applications where accuracy is paramount.

#### 3.2.2. Comparison of OA with Different SNR Levels

RadioML2016.10a and RadioML2016.10b include modulated signals with SNR levels from −20 dB to 18 dB. We categorized them into three intervals for analysis: extremely low SNR (−20 dB to −10 dB), low SNR (−8 dB to 0 dB), and high SNR (2 dB to 18 dB). In the extremely low SNR interval, noise is 10 to 100 times stronger than the signal, making feature extraction hard. The low SNR range, where noise power is about 1 to 6.3 times the signal power, also presents challenges in feature extraction. The high SNR interval, where signal power exceeds noise, supports higher classification accuracy.

**Figure 5:**
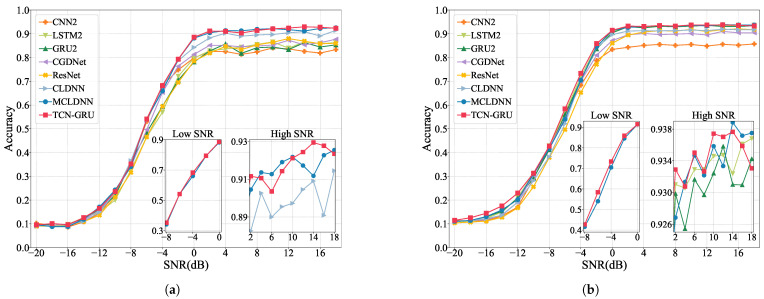
Overall accuracy of models at each SNR level. (**a**) RadioML2016.10a; (**b**) RadioML2016.10b.

Figure 5 provides a detailed OA overview of TCN-GRU other models across various SNR levels. The figure includes separate subgraphs to highlight the comparison between TCN-GRU and the high-performing MCLDNN model in the low SNR interval and the accuracy lines of several high accuracy networks in the high SNR interval. Specifically, for RadioML2016.10a in Figure 5a, our model demonstrates an OA improvement over MCLDNN of approximately 0.244%, 0.753%, and 0.192% in the extremely low, low, and high SNR intervals, respectively. Notably, accuracy is increased at the −4 dB and 14 dB SNR levels, with about 2.194% and 1.764%. For RadioML2016.10b in Figure 5b, the OA improvements of TCN-GRU are about 1.276%, 1.939%, and 0.052% across the three SNR intervals, with gains of approximately 4.367% and 2.861% at -6 dB and −4 dB, respectively, compared to MCLDNN. These results indicate that the TCN-GRU model’s robustness and effectiveness in challenging SNR scenarios outperform current models in AMC.

**Figure 6:**
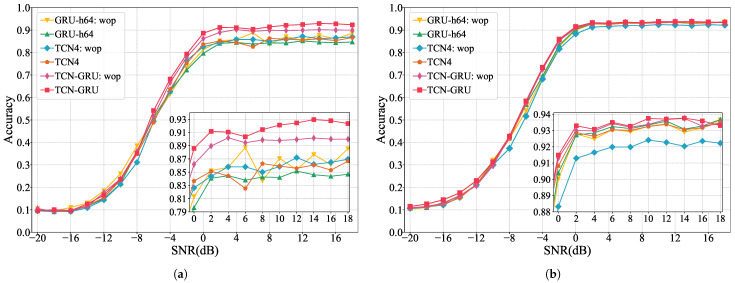
Overall accuracy of controlled models at each SNR level. (**a**) RadioML2016.10a; (**b**) RadioML2016.10b.

Figure 6 presents a line chart of the OA comparison between TCN-GRU and control group models at each SNR level from the ablation experiment. TCN-GRU consistently achieves the best recognition performance in both datasets, indicating that the combination of TCN, GRU, and GAP components is synergistic and effective.

#### 3.2.3. Comparison of the Recognition Accuracy of Different Modulated Signals

**Figure 7:**
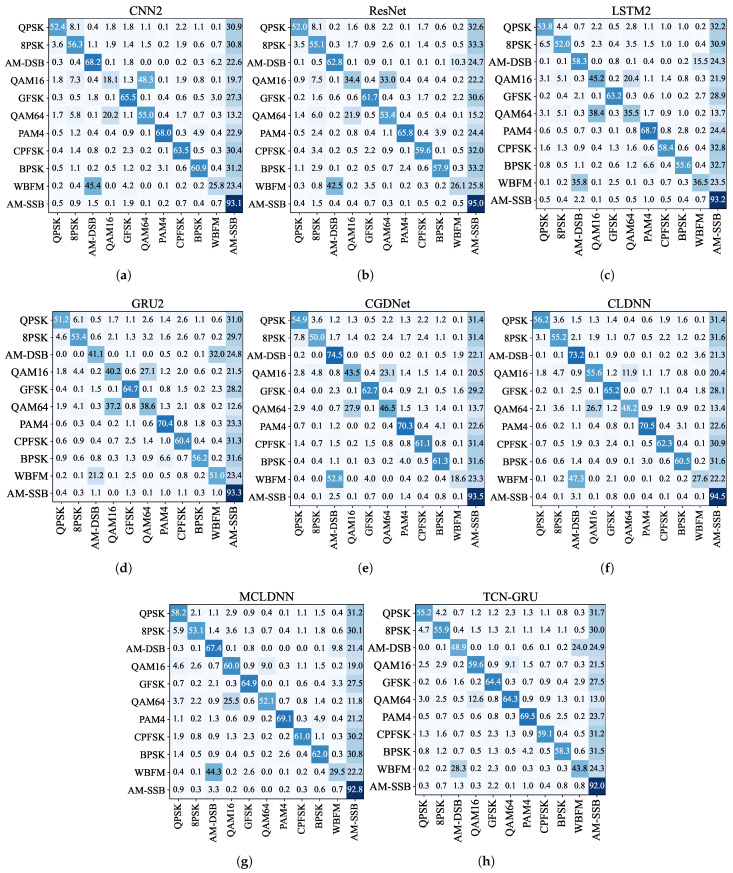
Confusion matrices for RadioML2016.10a. (**a**) CNN2; (**b**) ResNet; (**c**) LSTM2; (**d**) GRU2; (**e**) CGDNet; (**f**) CLDNN; (**g**) MCLDNN; (**h**) TCN-GRU. The color depth proportional to the predicted percentage of the corresponding category.

**Figure 8:**
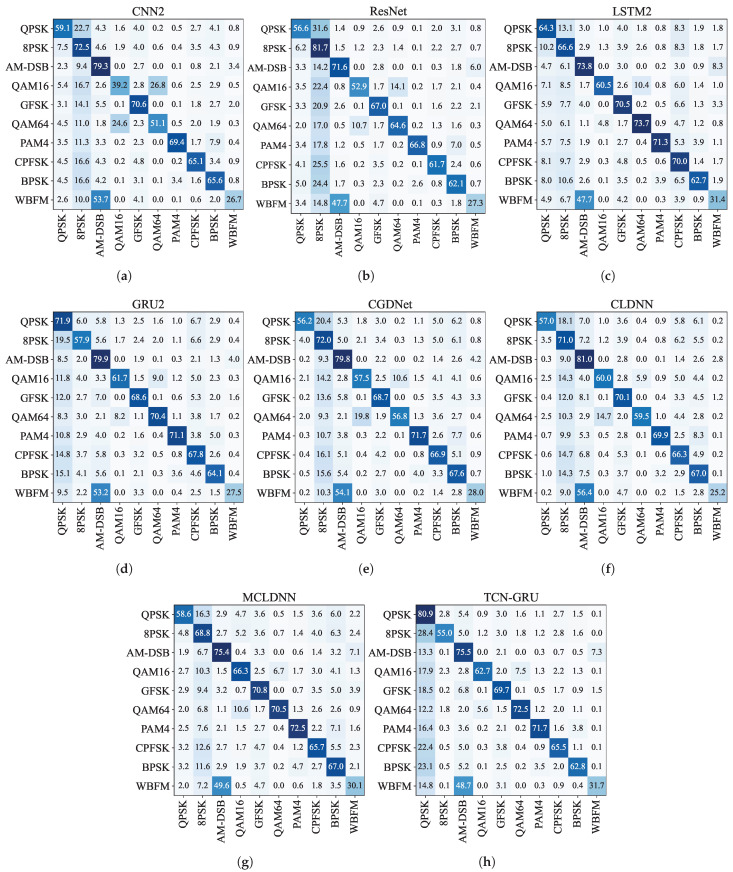
Confusion matrices for RadioML2016.10b. (**a**) CNN2; (**b**) ResNet; (**c**) LSTM2; (**d**) GRU2; (**e**) CGDNet; (**f**) CLDNN; (**g**) MCLDNN; (**h**) TCN-GRU. The color depth proportional to the predicted percentage of the corresponding category.

We use confusion matrices to illustrate the accuracy of models for different signal modulations. Figure 7 shows the results for all models on RadioML2016.10a, while Figure 8 presents the results for RadioML2016.10b. These matrices visually represent each model’s strengths and weaknesses in accurately identifying and classifying modulated signals across categories.

For RadioML2016.10a and RadioML2016.10b, three signal pairs are commonly confused: QPSK/8PSK, QAM16/QAM64, and AM-DSB/WBFM. QPSK and 8PSK, both phase modulation techniques, share sine and cosine oscillation characteristics, differing mainly in the number of phases. BPSK, with simpler phase changes, is less frequently confused with these modulations. QAM16 and QAM64, as quadrature amplitude modulations, use different amplitude and phase combinations to represent symbols, making them prone to misidentification. AM-DSB and WBFM are analog modulations. They share similarities in amplitude, leading to potential confusion.

TCN-GRU improves discrimination between QAM16 and QAM64 on both datasets and ranks high in distinguishing QPSK and 8PSK. Regarding AM-DSB and WBFM, the TCN-GRU model shows better discrimination ability on RadioML2016.10b, though it performs slightly less effectively on RadioML2016.10a.

Uniform distribution confusions in the confusion matrices, such as AM-SSB for RadioML2016.10a and 8PSK and QPSK for RadioML2016.10b, were observed across all models. This occurs when the signal is submerged in noise at extremely low SNR, preventing effective recognition. Models tend to select the modulation mode most similar to noise as the most likely outcome. Using RadioML2016.10a, AM-SSB resembles white noise across various SNRs, leading to the misidentification signals in extremely low SNR as AM-SSB, thus creating a uniform confusion pattern in the AM-SSB column. Consequently, AM-SSB achieves high recognition accuracy at all SNR levels. Similarly, using RadioML2016.10b, where AM-SSB is absent, signals in extremely low SNR are often misclassified as QPSK or 8PSK.

Due to high-power noise interference, all models show the aforementioned errors in the extremely low SNR interval, which can obscure their true capabilities. To address this, we excluded extremely low SNR signals from the test set, focusing on recognition performance from −8 dB to 18 dB in terms of accuracy. Table 5 and Table 6 show the recognition accuracies on the RadioML2016.10a and RadioML2016.10b datasets with extremely low SNR signals removed, providing a clearer assessment of the performance of each model over the range of SNRs in which most communication devices can work properly.

On RadioML2016.10a, our model significantly improves the discrimination ability of QPSK/8PSK and QAM16/QAM64 compared with other models. It also achieves a high OA for AM-DSB/WBFM, with an average accuracy of 0.6675. This performance is surpassed only by ResNet (0.6995). For other modulations, like PAM4, CPFSK, BPSK, GFSK, and AM-SSB, differences in classification accuracy across models are relatively small. On RadioML2016.10b, our model is also improved in classifying QAM16/QAM64 over other models. The OA for QPSK/8PSK and AM-DSB/WBFM are 0.8395 and 0.6725 higher, respectively, than MCLDNN’s accuracies of 0.8160 and 0.6645. Model performance is similarly competitive for PAM4, CPFSK, BPSK, and GFSK. These results highlight the TCN-GRU model’s enhanced capability to accurately identify specific modulated signals, particularly where other models may struggle.

## 4. Conclusions

In this study, we introduced a novel hybrid model TCN-GRU that combines three modules: TCN, GRU, and GAP. TCN-GRU has the characteristics of multiscale feature extraction, hybrid feature fusion, and lightweight model construction. It has the ability to improve the drawbacks of existing hybrid models in low recognition accuracy of complex modulated signals and excessive parameters. Experimental results on the RadioML2016.10a and RadioML2016.10b datasets highlight the model’s superior performance, achieving an average recognition accuracy of 0.6156 and 0.6466, respectively. In particular, TCN-GRU demonstrated a notable improvement in recognition accuracy for challenging modulations. Using RadioML2016.10a, TCN-GRU enhanced the differentiation of QAM16/QAM64 by approximately 10.5% over MCLDNN, while achieving similar performance in distinguishing QPSK/8PSK. Using RadioML2016.10b, TCN-GRU improved differentiation accuracy by approximately 6.7% for QPSK/8PSK and 1.6% for AMDSB/WBFM, while maintaining comparable performance on QAM16/QAM64. This means that multiscale feature extraction can improve the network’s signal recognition ability, and will make our model valuable in some specific communication scenarios.

Achieving high-precision modulation classification under low SNR conditions remains a significant and challenging task in this field. Even the existing SOTA method (MCLDNN) achieves an overall accuracy of only 0.4163, 0.5403, and 0.7048 at −8 dB, −6 dB, and −4 dB, respectively, using RadioML2016.10b. Our model improves accuracy by 1.10%, 4.37%, and 2.86%, respectively, compared to MCLDNN, reaching 0.4274, 0.5840, and 0.7334. However, we recognize that there are still needs for further enhancement. Additionally, TCN-GRU is a lightweight network requiring fewer parameters than other hybrid models, which reduces computational demands and lowers the risk of overfitting, thereby enhancing its generalization capability and adaptability for real-world deployment.

In future work, we will focus on the current limitations of TCN-GRU, using methods such as model optimization, integration of advanced denoising techniques, and transfer learning to enhance our model. These enhancements are designed to improve inference speed and classification accuracy at low SNR, enabling the model to learn a wider variety of signals with fewer samples and achieve higher efficiency to better adapt to new wireless standards.

## Figures and Tables

**Table 1:** Details of the RadioML2016.10a and RadioML2016.10b datasets used in the experiment. RadioML2016.10a includes 11 types of modulated signals, while RadioML2016.10b has 10 types. Both datasets cover an SNR range from −20 dB to 18 dB in 2 dB increments.

Dataset	Sample	SNR	Modulation Types
RadioML2016.10a	220,000	−20:2:18 dB	BPSK, QPSK, 8PSK, QAM16, QAM64, GFSK, CPFSK, PAM4, WBFM, AM-SSB, AM-DSB
RadioML2016.10b	1,200,000	−20:2:18 dB	BPSK, QPSK, 8PSK, QAM16, QAM64, GFSK, CPFSK, PAM4, WBFM, AM-DSB

**Table 2:** The specific structure of each model, including the CNN models, the RNN models, the hybrid models, and the control group models for the ablation experiment.

Model	Structure
CNN2 [18]	I/Q-Input, Conv1(c:64,k:1×8), Conv2(c:16,k:2×8), FC1(128), FC2(*N*)
ResNet [19]	I/Q-Input, Conv1(c:256,k:1×3), Conv2(c:256,k:2×3), Concat(I/Q-Input, Conv3(c:80,k:13)), Conv4(c:80,k:13), FC1(128), FC2(*N*)
LSTM2 [22]	A/P-Input, LSTM(h:128) × 2, FC(*N*)
GRU2 [25]	I/Q-Input, GRU(h:128) × 2, FC(*N*)
CGDNet [35]	I/Q-Input, Conv1(c:50,k:1×6), MaxPool1(k:2×2), Conv2(c:50,k:1×6), MaxPool2(k:2×2), Conv3(c:50,k:1×6), MaxPool3(k:2×2), Concat with MaxPool1, GRU(h:50), FC1(256), FC2(*N*)
CLDNN [19]	I/Q-Input, Conv1(c:256,k:1×3), Conv2(c:256,k:2×3), Conv3(c:80,k:1×3) × 2, LSTM(h:50), FC1(128), FC2(*N*)
MCLDNN [23]	Concat1(I-Input, Conv1(c:50,k:1×8), Q-Input, Conv2(c:50,k:1×8)), I/Q-Input, Conv3(c:50,k:1×8), Concat2(Concat1, Conv4(c:50,k:2×8)), Conv5(c:100,k:2×8), LSTM(h:128), FC1(128), FC2(128), Softmax(*N*)
GRU2-h64: wop	A/P-Input, GRU(h:64) × 2, FC(*N*)
GRU2-h64	A/P-Input, GRU(h:64) × 2, GAP, FC(*N*)
TCN4: wop	A/P-Input, TCN ResBlock(h:64,k:7) × 4, FC(*N*)
TCN4	A/P-Input, TCN ResBlock(h:64,k:7) × 4, GAP, FC(*N*)
TCN-GRU: wop	A/P-Input, TCN ResBlock(h:64,k:7) × 4, GRU(h:64) × 2, FC(*N*)
TCN-GRU	A/P-Input, TCN ResBlock(h:64,k:7) × 4, GRU(h:64) × 2, GAP, FC(*N*)

**Table 3:** Comparison of parameters, performance evaluation indicators, and prediction time among models for the RadioML2016.10a dataset. It should be noted that the data types of RadioML2016.10a are uniformly distributed, which makes Accuracy and Recall equal in value.

Model	Parameters	Valid Loss	Accuracy	F1-Score	Recall	Precision	Prediction Time (ms)
CNN2	1,592,383	1.1434	0.5698	0.5819	0.5698	0.6733	43.7356
ResNet	3,098,283	1.1809	0.5638	0.5861	0.5638	0.6891	61.7528
LSTM2	201,099	1.1196	0.5849	0.6033	0.5849	0.6945	29.2208
GRU2	151,179	1.1292	0.5692	0.5866	0.5692	0.6801	25.2324
CGDNet	124,933	1.1288	0.5789	0.5935	0.5789	0.7059	21.1059
CLDNN	517,643	1.1002	0.6056	0.6275	0.6056	0.7206	64.4990
MCLDNN	406,199	1.0824	0.6101	0.6344	0.6101	0.7321	128.4710
GRU2-h64: wop	38,731	1.1326	0.5708	0.5885	0.5708	0.6787	18.0023
GRU2-h64	38,731	1.1074	0.5916	0.6077	0.5897	0.6834	17.4842
TCN4: wop	203,531	1.1285	0.5758	0.5945	0.5738	0.7031	113.3784
TCN4	203,660	1.1174	0.5820	0.6034	0.5820	0.7018	116.3363
TCN-GRU: wop	253,451	1.0914	0.6143	0.6278	0.6085	0.7199	132.2325
TCN-GRU	253,451	1.0797	0.6156	0.6389	0.6156	0.7400	136.1084

**Table 4:** Similar to Table 3, but using the RadioML2016.10b dataset.

Model	Parameters	Valid Loss	Accuracy	F1-Score	Recall	Precision	Prediction Time (ms)
CNN2	1,592,126	0.9781	0.5944	0.5930	0.5944	0.6402	229.6624
ResNet	3,098,154	0.9631	0.6123	0.6259	0.6123	0.7001	325.3973
LSTM2	200,970	0.8818	0.6442	0.6448	0.6442	0.6900	148.6904
GRU2	151,050	0.8965	0.6393	0.6439	0.6393	0.7096	130.3700
CGDNet	124,676	0.9386	0.6217	0.6255	0.6217	0.6785	114.5280
CLDNN	517,514	0.9366	0.6269	0.6306	0.6269	0.7014	346.8208
MCLDNN	406,070	0.8864	0.6462	0.6444	0.6462	0.6761	705.3022
GRU2-h64: wop	38,666	0.8985	0.6402	0.6403	0.6386	0.6913	103.8465
GRU2-h64	38,666	0.8936	0.6398	0.6447	0.6392	0.7172	106.9712
TCN4: wop	203,595	0.9239	0.6273	0.6282	0.6268	0.6801	630.0027
TCN4	203,466	0.8842	0.6431	0.6477	0.6431	0.7066	635.1041
TCN-GRU: wop	253,386	0.8824	0.6445	0.6531	0.6439	0.7254	732.0409
TCN-GRU	253,386	0.8755	0.6466	0.6591	0.6466	0.7536	743.2286

**Table 5:** Accuracy of each modulation in low SNR and high SNR interval (−8 dB to 18 dB), RadioML2016.10a.

Model	QPSK	8PSK	AM-DSB	WBFM	QAM16	QAM64	PAM4	CPFSK	BPSK	GFSK	AM-SSB
CNN2	0.785	0.818	0.903	0.379	0.456	0.886	0.960	0.862	0.872	0.859	0.937
ResNet	0.796	0.822	0.942	0.457	0.592	0.770	0.971	0.926	0.924	0.960	0.950
LSTM2	0.803	0.761	0.801	0.518	0.692	0.464	0.971	0.874	0.850	0.906	0.950
GRU2	0.793	0.789	0.702	0.588	0.625	0.497	0.968	0.889	0.864	0.923	0.943
CGDNet	0.801	0.737	0.967	0.276	0.639	0.613	0.959	0.880	0.871	0.905	0.951
CLDNN	0.801	0.800	0.936	0.371	0.742	0.641	0.956	0.882	0.857	0.920	0.940
MCLDNN	0.851	0.786	0.896	0.436	0.856	0.744	0.976	0.883	0.901	0.941	0.936
TCN-GRU	0.859	0.828	0.718	0.617	0.877	0.901	0.979	0.879	0.884	0.924	0.932

**Table 6:** Accuracy of each modulation in low SNR and high SNR interval (−8 dB to 18 dB), RadioML2016.10b.

Model	QPSK	8PSK	AM-DSB	WBFM	QAM16	QAM64	PAM4	CPFSK	BPSK	GFSK
CNN2	0.817	0.866	0.972	0.362	0.553	0.710	0.969	0.929	0.894	0.947
ResNet	0.790	0.921	0.950	0.375	0.775	0.884	0.960	0.904	0.893	0.945
LSTM2	0.839	0.823	0.944	0.413	0.864	0.960	0.974	0.933	0.882	0.946
GRU2	0.858	0.789	0.958	0.388	0.881	0.936	0.973	0.931	0.885	0.943
CGDNet	0.798	0.818	0.953	0.371	0.789	0.774	0.971	0.923	0.892	0.927
CLDNN	0.809	0.822	0.949	0.354	0.835	0.810	0.957	0.915	0.885	0.937
MCLDNN	0.798	0.834	0.952	0.377	0.890	0.951	0.973	0.910	0.875	0.944
TCN-GRU	0.881	0.798	0.893	0.452	0.893	0.962	0.975	0.927	0.888	0.952

## Data Availability

Data are contained within the article.
